# DHA-Enriched Fish Oil Ameliorates Deficits in Cognition Associated with Menopause and the *APOE4* Genotype in Rodents

**DOI:** 10.3390/nu14091698

**Published:** 2022-04-19

**Authors:** Matthew G. Pontifex, Anneloes Martinsen, Rasha N. M. Saleh, Glenn Harden, Chris Fox, Michael Muller, David Vauzour, Anne-Marie Minihane

**Affiliations:** 1 Norwich Medical School, University of East Anglia, Norwich NR4 7TJ, UK; a.martinsen@uea.ac.uk (A.M.); r.saleh@uea.ac.uk (R.N.M.S.); g.harden@uea.ac.uk (G.H.); chris.fox@uea.ac.uk (C.F.); michael.muller@uea.ac.uk (M.M.); d.vauzour@uea.ac.uk (D.V.); a.minihane@uea.ac.uk (A.-M.M.); 2 Clinical Pathology Department, Faculty of Medicine, Alexandria University, Alexandria 21526, Egypt; 3 Exeter Medical School, University of Exeter, Exeter EX4 4PY, UK

**Keywords:** Alzheimer’s disease, apolipoprotein E, arachidonic acid, docosahexaenoic acid, brain, BDNF, oestrogen, oestrogen receptor, Glut-5, 4-vinylcyclohexanediepoxide

## Abstract

Female *APOE4* carriers have a greater predisposition to developing Alzheimer’s disease (AD) compared to their male counterparts, which may partly be attributed to menopause. We previously reported that a combination of menopause and *APOE4* led to an exacerbation of cognitive and neurological deficits, which were associated with reduced brain DHA and DHA:AA ratio. Here, we explored whether DHA-enriched fish oil (FO) supplementation mitigated the detrimental impact of these risk factors. Whilst DHA-enriched fish oil improved recognition memory (NOR) in *APOE4* VCD (4-vinylcyclohexene diepoxide)-treated mice (*p* < 0.05), no change in spatial working memory (Y-maze) was observed. FO supplementation increased brain DHA and nervonic acid and the DHA:AA ratio. The response of key bioenergetic and blood–brain barrier related genes and proteins provided mechanistic insights into these behavioural findings, with increased BDNF protein concentration as well as mitigation of aberrant *Erβ*, *Cldn1* and *Glut*-*5* expression in *APOE4* mice receiving fish oil supplementation (*p* < 0.05). In conclusion, supplementation with a physiologically relevant dose of DHA-enriched fish oil appears to offer protection against the detrimental effects of menopause, particularly in “at-risk” *APOE4* female carriers.

## 1. Introduction

Developing interventions to prevent or delay onset of dementia is a key priority [1]. *APOE4* is the strongest common genetic risk factor for the development of Alzheimer’s disease (AD) [2]; however, a fundamental understanding of its role in AD has been confounded by the pleiotropic nature of the *APOE* gene [3]. Evidence suggests that the AD risk associated with an *APOE4* genotype is greater in females [4,5], particularly between the ages of 55 and 70 years [6], which may partly account for the greater overall incidence of AD in women [7]. The specific age at which this heightened risk occurs, coupled with the importance of oestrogens in cognition and the association between menopause and cognitive decline, is indicative of a potential menopausal involvement [8,9]. From the limited evidence currently available [10,11,12,13], neurological deficits associated with menopause may be exacerbated by an *APOE4* genotype.

Docosahexaenoic acid (DHA) is the primary long-chain n-3 PUFA present in the brain, accounting for ~15% of total lipids [14]. Epidemiological studies have shown that higher DHA intake and status, primarily achieved through intake of oily fish, improves cognitive performance and reduces AD risk [15,16,17]. Although evidence from randomised control trials with DHA supplementation has been inconsistent [18,19,20], potentially due to variability in study designs (e.g., length of intervention, stage of disease progression and dose of intervention and DHA:EPA ratio), the benefits of DHA supplementation have been widely reported in cells and rodent models of AD [21,22,23]. Such models have revealed several brain structural and functional roles for DHA [24], including neuronal signalling, survival/neurogenesis, anti-amyloidogenic and anti-inflammatory effects [24,25,26]. In addition, human studies utilising ^11^C- and ^13^C-labelled carbon have identified *APOE4*-associated deficits in DHA metabolism and transport [27,28], which may account for *APOE4*-associated lower brain DHA status as we had previously reported [29]. As a result, *APOE4* carriers may be more vulnerable to dietary n-3 PUFA deficiencies [30], which may in turn impact cognition, thus providing a rationale for higher intake [31]. There is evidence that both menopause and a high-fat (HF) diet disrupt n-3 PUFA status [32,33,34,35]. Therefore, postmenopausal *APOE4* carriers consuming “Western-style” diets may be at particular risk of DHA deficiency and are likely to be responsive to intervention. We had previously reported that both *APOE4* and menopause impact cognition, synaptic plasticity and brain DHA [13]. Here, we posit that supplementation with DHA-rich fish oil (FO) may restore DHA status and in turn ameliorate the cognitive deficits observed in postmenopausal *APOE4* carriers. The effects of DHA supplementation were established by administering a physiologically relevant concentration of DHA-enriched FO to an *APOE*-TR mouse model where human menopause was induced by 4-vinylcyclohexane diepoxide (VCD) treatment. Behavioural tests of cognition were performed and were related to brain fatty acid, protein and gene expression profiles.

## 2. Materials and Methods

### 2.1. Study Approval

All experimental procedures and protocols used in this study were reviewed and approved by the Animal Welfare and Ethical Review Body (AWERB) and were conducted within the provisions of the Home Office Animals (Scientific Procedures) Act 1986.

### 2.2. Animal Model and Experimental Design

Female humanised *APOE3* (B6.129P2-Apoe^tm2(APOE*3)Mae^ N8)- and *APOE4* (B6.129P2-Apoe^tm2(APOE*4)Mae^ N8)-targeted replacement mice homozygous for the human *APOE3* or *APOE4* gene (Taconic, Germantown, NY, USA) were used in these experiments [29,36,37]. Mice were maintained in a controlled environment (21 ± 2 °C; 12 h light–dark cycle; light from 07:00 h) and fed ad libitum on a standard chow diet (RM3-P, Special Diet Services, Essex, UK) until the age of 4 months, ensuring normal development. Following this run-in period, mice were switched to one of two diets (Research Diets, New Brunswick, NJ, USA) for the remaining experimentation. The two diets were as follows: (1) high-fat diet (45 kCal% from fat) (HF) (2) and high-fat diet (45 kCal% from fat) with the addition of DHA-enriched fish oil (HFFO) (see Table S1 for full dietary composition). A HF diet was chosen to mimic a human “Western-style” diet, a risk factor prevalent in modern society that exacerbates AD-like age-related cognitive decline [38]. For the fish-oil-enriched diet, a bespoke blend of EPAX 1050 TGN + EPAX 6000 TGN (4:1 DHA:EPA fish oil) gifted from Epax^®^ (Oslo, Norway) was added to the background diet. A 4:1 ratio (*w*/*w*) was selected based on previous studies [39], which state the importance of DHA in *APOE4* carriers and the fact that the brain is highly enriched in DHA relative to the EPA. However, some EPA was retained on the basis that human oily fish sources provide both EPA and DHA in variable ratios depending on species and that EPA is important for glial cell function [40]. The enriched diet was designed to provide DHA + EPA at 4.7 g/kg of diet. Given that mean food intake in the mice was 3.2 g/day, this equated to a DHA + EPA human dose of ~2 g per day based on allometric scaling and body surface (BSA)-based calculations [41,42]. To prevent lipid oxidation, diets were stored at −20 °C until use, and fresh feed was provided every 3 days.

Menopause was induced using 14 injections of VCD (160 mg/kg) over a 3-week period beginning at 8 months of age [13], resulting in menopause being induced at 12 months of age, which roughly corresponds to middle age for a C57BL/6 mouse. Following completion of the final behavioural test, animals aged 12 months were sedated with a mixture of isoflurane (1.5%) in nitrous oxide (70%) and oxygen (30%) and transcardially perfused with an ice-cold PBS containing protease (SIGMAFAST^TM^ protease inhibitor, Sigma, Devon, UK) and phosphatase (1 mM sodium pyrophosphate and 50 mM sodium fluoride, Sigma, Devon, UK) inhibitors. Sera were isolated via centrifugation at 2000× *g* for 10 min. Brains were rapidly removed, halved, snap frozen and stored at −80 °C until biochemical analysis. Animal numbers at study completion were as follows: *APOE3* HF VCD, *n* = 12; *APOE4* HF VCD, *n* = 12; *APOE3* HF FO, *n* = 10; *APOE4* HF FO, *n* = 7.

### 2.3. Behavioural Assessment

All behavioural tests were performed when mice reached 12 months of age. Prior to commencing, a visual placing test was performed on each animal to ensure animals were not visually impaired [43]. All behavioural tests were analysed using the Ethovision software (Tracksys Ltd., Nottingham, UK).

The novel object recognition (NOR), a measure of recognition memory was performed as described previously [44,45] with slight modifications. Briefly, on day 1, habituation was conducted in the empty maze for 10 min. On day 2, animals were conditioned to a single object for a 10 min period. On day 3, mice were exposed to 2 identical objects for 15 min. Following an intertrial interval of one hour, mice were placed back within the testing arena now containing one familiar object and one novel object. Videos were analysed for a 5 min period, after which if an accumulative object exportation of 8 s failed to be reached, analysis continued for the full 10 min or until 8 s was achieved. Those not achieving 8 s exploration were excluded from the analysis [46]. Preference index, the ratio of novel object exploration time divided by the total exploration time with both objects, was calculated.

The Y-maze spontaneous alternation test, a measure of spatial working memory, was performed as previously described [13]. Ethovision software was used to analyse each animal for 7 min, with zone transitioning and locomotor activity recorded. Spontaneous alternation was calculated using the following formula: (number of alternations/max number of alternations × 100).

### 2.4. Biochemical Analyses

Follicle-stimulating hormone (FSH) and brain-derived neurotrophic factor (BDNF) concentrations were determined by ELISA (Abnova, Taipei, Taiwan, ref KA2330 and R&D Systems, Minneapolis, MN, USA, ref DY248, respectively) in sera samples and cerebral cortex tissue, respectively, as per the manufacturer’s instructions. Total lipids were extracted from subcortical brain tissues (*n* = 5/6 per group), and the erythrocyte fraction using the Folch extraction method [47] as previously reported [29].

### 2.5. RNA Isolation and qRT-PCR

RNA isolation, cDNA synthesis and RT-qPCR were carried out as previously described [48]. Briefly, total RNA was isolated from the hippocampal samples using the Qiazol reagent (Qiagen, Manchester, UK). Here, 1 μg of total RNA was treated with DNase I (Invitrogen, Renfrew, UK) and used for cDNA synthesis using Invitrogen™ Oligo (dT) primers and M-MMLV reverse transcriptase. Quantitative real-time PCR (RT-qPCR) reactions were performed using SYBR green detection technology on the Roche light cycler 480 (Roche Life Science, Penzberg, Germany). Results are expressed as relative quantity scaled to the average across all samples per target gene and normalised to the reference gene glyceraldehyde-3-phosphate dehydrogenase (*Gapdh*), which was identified as the optimal housekeeping selection/combination using the Normfinder software [49]. The primer sequences are given in Table S2.

### 2.6. Statistical Analysis

All data are presented as mean ± SEM. Data analysis was performed in GraphPad Prism version 8 (GraphPad Software, San Diego, CA, USA). After checking for normality/equal variances and performing transformation where necessary, comparisons among groups were performed using either two-way or three-way ANOVA examining the impact of FO intervention and genotype (*APOE3* and *APOE4*) as well as age (in months for body weight analysis) on the outcome variables. The *p* values were corrected for multiple testing using the Benjamini–Hochberg false discovery rate (FDR), with FDR 5% applied for comparison; *p* values of less than 0.05 were considered statistically significant.

## 3. Results

### 3.1. FO Supplementation or Ovarian Failure Does Not Significantly Modify Weight Gain

**Figure 1:**
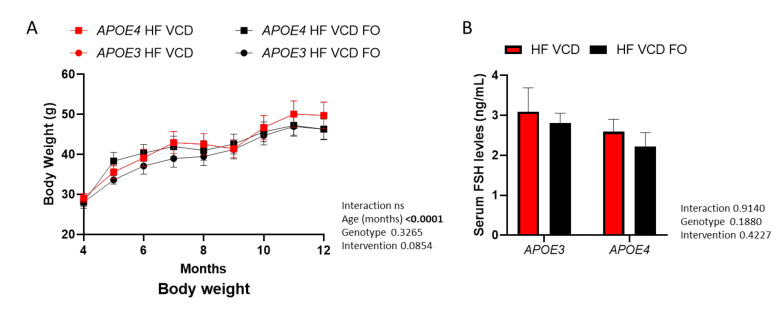
Effects of fish oil (FO) supplementation on body weight and follicle-stimulating hormone (FSH) levels. (**A**) Although body weight significantly increased as animals aged, it was not altered across genotype or FO supplementation (three-way ANOVA *n* ≥ 7). (**B**) Serum FSH levels remained unchanged by FO intervention. Data are presented as mean ± SEM.

The results included here are part of a study series with our previous report, which detailed the impact of VCD treatment on phenotype relative to sham-injected animals [13]. The data for our reference VCD-treated animals have been previously reported [13]. Although body weight significantly increased over the 8-month intervention (*p* < 0.0001; Figure 1A), it did not differ across experimental groups. The effect of VCD treatment on serum FSH levels (see [13]) was not altered by FO intervention (*p* > 0.05; Figure 1B).

### 3.2. DHA-Rich FO Supplementation Restores APOE4-Induced Impairment in Recognition Memory

**Figure 2:**
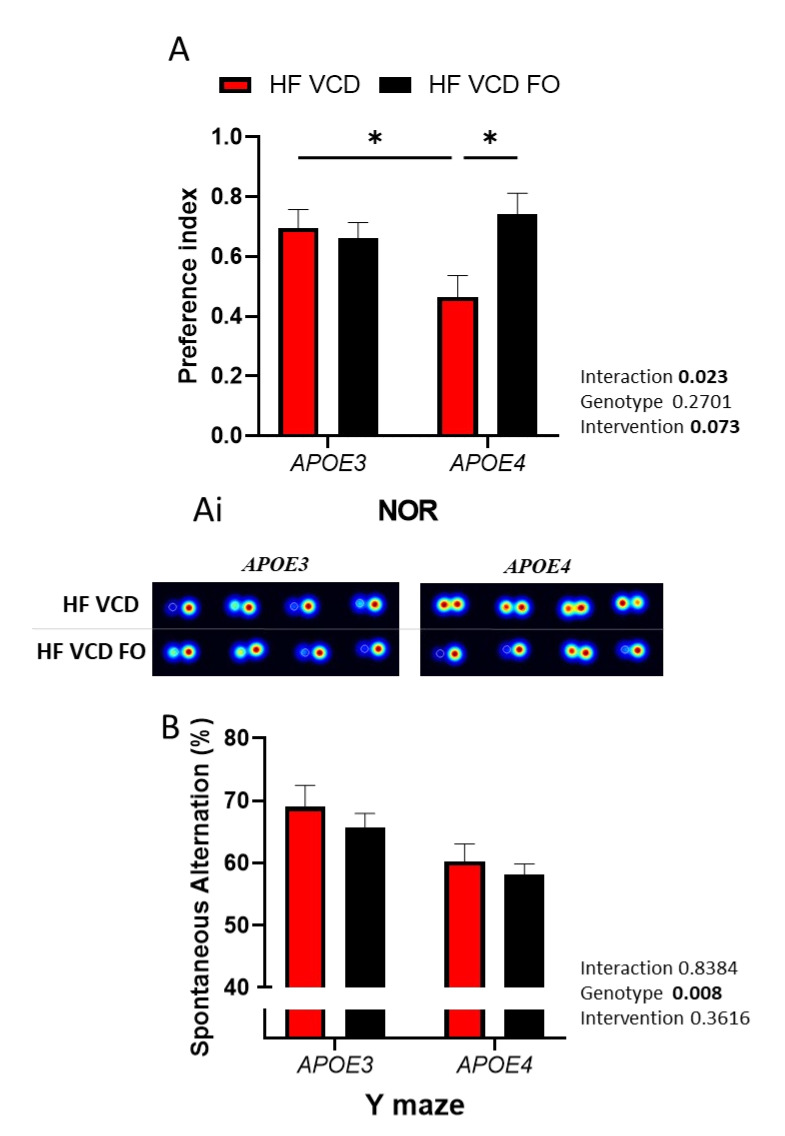
The effect of fish oil (FO) supplementation on cognitive performance: (**A**) Recognition memory was improved via FO supplementation (*n* ≥ 6); (**Ai**) representative heatmaps of the performance of individual animals. (**B**) The Y-maze performance diminished under *APOE4* genotype and was not influenced by FO supplementation. Data are presented as mean ± SEM; * *p* < 0.05.

As previously reported, VCD treatment was detrimental to recognition memory performance in *APOE4* animals [13]. Interestingly, supplementation with DHA-enriched FO improved recognition memory performance by 37% (*p* < 0.05; Figure 2A, with representative heatmaps shown in Figure 2Ai). Object recognition was not influenced by changes in locomotor activity (Figure S1A,B).

Conversely, the Y-maze performance, a measure of spatial working memory, was reduced as a result of *APOE4* genotype (*p* < 0.01) and was not improved by FO supplementation (Figure 2B).

### 3.3. DHA-Rich FO Supplementation Increases DHA Levels in the Brain of VCD-Treated Animals

FO supplementation significantly increased brain DHA levels (*p* < 0.01; Table 1). Furthermore, brain DHA:AA ratio, posited as an indicator of anti-inflammatory status, significantly increased in response to supplementation, with levels across both genotypes increasing by 30% (*p* < 0.0001; Table 1). In addition to n-3 PUFA status, changes in n-6 PUFA status were also apparent (Table 1). Furthermore, individual monounsaturated (MUFA) and saturated fatty acids (SFA) were modulated by FO intervention (Table S3), indicating broader fatty acid changes. Notably, levels of nervonic acid (24:1 n-9) which were diminished in *APOE4* VCD-treated animals (reference), increased through FO supplementation (*p* < 0.05; Table 1). 

### 3.4. FO Supplementation Improves Brain Deficits Induced by VCD and APOE4 Genotype

**Figure 3:**
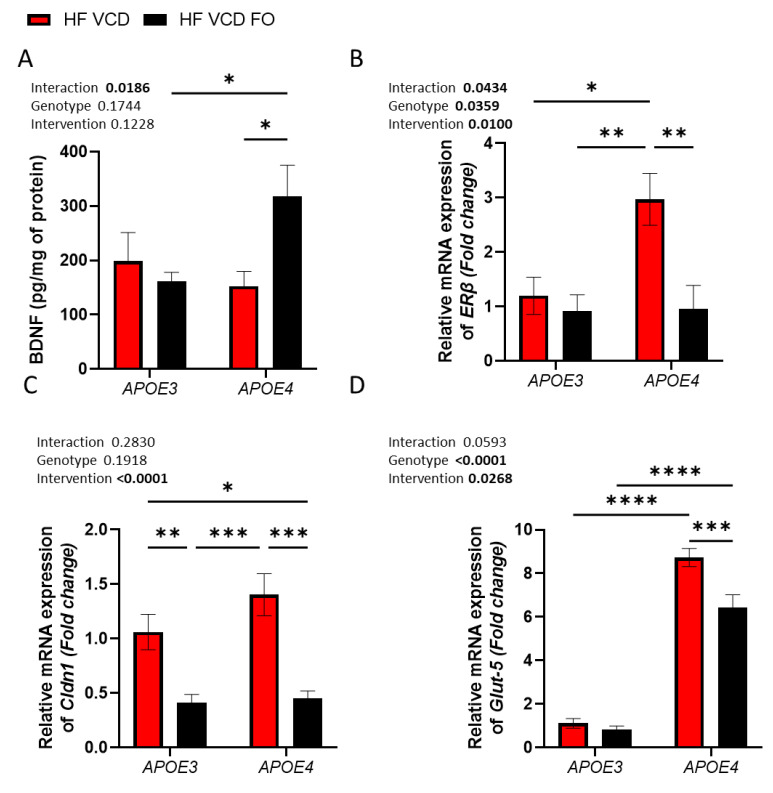
The effect of fish oil (FO) supplementation on molecular targets in *APOE4* and VCD-induced dysregulation. (**A**) Addition of FO supplementation increased BDNF levels in only *APOE4* animals. (**B**) Increased Erβ expression observed in *APOE4* VCD-treated animals was mitigated by FO supplementation. (**C**) *Cldn1* was upregulated by VCD treatment; this expression was reduced across both genotypes through FO supplementation. (**D**) Expression of the fructose transporter *Glut*-*5* was considerably increased in *APOE4* animals compared to *APOE3* and was partially recovered by FO treatment. Data are presented as mean ± SEM; * *p* < 0.05, ** *p* < 0.01, *** *p* < 0.001, **** *p* < 0.0001.

In *APOE4* animals, FO supplementation resulted in a 2-fold increase in BDNF concentration (*p* < 0.05; Figure 3A). Expression of the oestrogenic receptor *ERβ* was distinctly increased in *APOE4* VCD-treated animals, with the expression 2.5-fold greater than in *APOE3* VCD-treated animals (*p* < 0.05; Figure 3B). The addition of FO prevented this change associated with *APOE4* and VCD (*p* < 0.01; Figure 3B). Gene expression of the tight junction proteins *Cldn1* and *ZO*-*1* were similarly reduced by FO treatment, although only *Cldn1* achieved significance (*p* < 0.0001 and *p* = 0.0526; Figure 3C and Table 2, respectively). Finally, we observed a large change in the expression of bioenergetic/metabolic-related genes (predominantly associated with fructose metabolism) within the brain. Notably, expression of the fructose transporter *Glut*-*5* was increased 8-fold in *APOE4* animals (*p* < 0.0001; Figure 3D). FO treatment significantly reduced *Glut*-*5* expression by 14% compared to *APOE4* VCD-treated animals (*p* < 0.001 Figure 3D). In contrast, *Glut*-*1* remained constant, whilst *Glut*-*3* was reduced in response to FO supplementation (*p* < 0.05, Table 2). *Chrebp* and *Gsk3b* were both similarly reduced by FO supplementation (*p* < 0.01, Table 2). Interestingly, the fructose-metabolising enzyme *Aldob* was expressed to a greater extent in *APOE4* animals (*p* < 0.05; Table 2). As with *Gsk3b* and *Chrebp*, FO supplementation reduced *Aldob* expression (*p* < 0.05; Table 2). We observed no change in *ERα* (Table 2).

## 4. Discussion

*APOE4* is pleiotropic and acts upon multiple physiological processes and pathological cascades [50]. The purported ability of DHA to modulate several of these pathways emphasises the potential of DHA as a lifestyle strategy to mitigate *APOE4*-associated AD risk through incorporation into the diet [51]. The present study follows on from our previous report, in which both *APOE4*- and VCD-mediated deficits in cognition were established [13]. Here, we showed that supplementation with DHA-enriched fish oil at a physiologically relevant dose improves recognition memory deficits in *APOE4* VCD-treated animals. Gene expression and protein analysis supported these behavioural findings, with DHA-enriched fish oil supplementation increasing BDNF protein abundance and ameliorating tight junction and metabolic gene disturbances. Furthermore, brain DHA was increased by DHA-enriched fish oil supplementation for both genotypes, as was the DHA:AA ratio, both of which were diminished in *APOE4* VCD-treated animals.

DHA-enriched fish oil supplementation mitigated the diminished recognition memory induced by the menopause mimic specifically in *APOE4*. There are limited reports evaluating the cognitive impact of n-3 PUFA supplementation in menopausal models. To the best of our knowledge, this is the first report in an *APOE*-TR menopause model and according to *APOE* genotype status. We previously identified reduced Y-maze and Barnes maze performance in *APOE4* animals [13], indicating a genotype-mediated spatial memory impairment that is independent of VCD treatment and that may be sex-specific [52]. The absence of VCD influence may indicate a predisposition in middle-aged *APOE4* females, which is uncoupled from menopause [52], although sex hormones may still be involved. The deficits in spatial working memory as assessed through Y-maze were not improved by FO supplementation, indicating subtleties between brain regions in their sensitivity to DHA supplementation. Supplementation in *APOE3* animals had no effect on cognition, likely due to the lack of impairment in these animals [39]. This may change throughout the ageing process as they become more susceptible to cognitive decline associated with ageing. Utilising specific “brain-targeting” DHA (i.e., in phospholipid form), which is metabolised to LPC-DHA and reportedly more efficacious at crossing the blood–brain barrier (BBB) [53], may allow lower dose supplementation. Interestingly, Sugasini and colleagues reported an improvement in *APOE4*-mediated spatial memory deficits when utilising 45 mg DHA/kg LPC-DHA, further supporting this notion [54].

DHA-enriched fish oil increased brain DHA levels in both *APOE3* and *APOE4* VCD-treated animals. Allessandri and colleagues [55] observed that reduction in brain DHA resulting from ovariectomy was restored through 17β-estradiol (E2) treatment. Interestingly, they reported that 17β-estradiol treatment increased both hepatic and brain LC-PUFA-synthesising enzymes, such as Δ9-, Δ6- and Δ5-desaturase, restoring or maintaining brain DHA. Supplementation with DHA-rich n-3 PUFA in this study may similarly restore [56] or compensate for the dysregulation of these synthesising enzymes, subsequently improving the brain PUFA profile. It has been reported that transport of DHA into the brain (as free fatty acid) was impaired in humans and 13-month-old *APOE4-TR* mice (only when compared to *APOE2* mice) [27,57]. However, in our model, DHA supplementation between 4 and 12 months increased DHA in a genotype-independent manner, suggesting that significant deficits in brain DHA uptake are not yet apparent at middle age. This is consistent with the findings of Yassine and colleagues, who conducted a PET study with [1-(11)C]-DHA and actually observed a higher DHA incorporation coefficient in several brain regions in middle-aged *APOE4* carriers, with the authors concluding that this may compensate for higher *APOE4*-associated DHA turnover [28]. In addition, the dysregulated fatty acid profile caused by menopause reportedly leads to alterations in neuronal membrane lipid raft structure, which can in turn disrupt the signalosome [58]. Supplementation with DHA could mitigate such detrimental effects. Furthermore, the DHA:AA ratio in the brain improved through fish oil supplementation, which is in line with other reports [59,60]. The dysregulation and subsequent restoration of FA homeostasis may be partly responsible for the cognitive profile observed. Indeed, DHA:AA has been used to predict mild cognitive impairment/AD development [61], with alterations in this ratio profoundly impacting inflammation [29,60,62,63]. However, both *APOE3* and *APOE4* animals exhibited similar PUFA dysregulation in response to VCD and as such cannot completely explain the cognitive data. Further exploration is therefore warranted. Individual SFAs and MUFAs were altered by genotype and fish oil supplementation, highlighting broader fatty acid modulation. For example, the MUFA (nervonic acid) was increased in *APOE4* animals receiving DHA-enriched fish oil. Believed to be a neuroprotective mediator [64,65], this alteration demonstrates the wider influence of DHA on other lipid entities that may contribute to brain health.

As with cognitive performance and brain DHA status, fish oil supplementation improved BDNF protein levels. Surprisingly, this was restricted to *APOE4* animals, with the aetiology of the *APOE* genotype difference currently not understood. The neurological benefits of n-3 PUFA supplementation on the neurotrophic factor (BDNF) have been widely reported [66,67], and likely contributed to the improved cognitive function observed. Further gene expression analysis revealed changes in oestrogen receptors, tight junction proteins and metabolic gene profiles. Firstly, dysregulation of the oestrogen receptor ERβ was apparent in *APOE4* VCD-treated animals, with DHA-enriched fish oil supplementation mitigating this increase. Disturbances in oestrogen receptor signalling has been highly connected with brain ageing and neurodegenerative disease as these receptors influence several neural processes, including proliferation, neuroinflammation, cholesterol metabolism and synaptic plasticity [68]. Furthermore, oestrogen receptors may directly modulate *APOE* expression in the brain [69], which one might speculate could lead to the exacerbation of the *APOE4* genotype. Expression of the tight junction proteins *Cldn1* and *ZO*-*1* was reduced by FO supplementation. Interestingly, *Cldn1* has been reported to be expressed in pathological conditions, specifically during BBB leakage, and is an indicator of tight junction complex disorganization [70]. If so, it may be the case that menopause exacerbates BBB leakiness and should be the focus of future research endeavours. Conversely, the increased expression may indicate loss of protein and therefore increased expression to compensate for this. Nevertheless, DHA-enriched fish oil supplementation altered this gene expression, reaffirming the involvement of DHA in maintaining tight junction and potentially BBB integrity and function. Finally, FO treatment altered metabolic gene expression in the *APOE*-*TR* VCD model. Most notably, the dramatic increase in *Glut*-*5* expression in *APOE4* animals was partially mitigated by FO treatment. To our knowledge, there are no reports currently highlighting such an interaction. We therefore speculate that this may indicate impaired glucose metabolism and a subsequent switch to the use of fructose. This is potentially supported by the concomitant changes in *Aldob*, *Chrebp* and *Gsk3b*, which further support the notion that DHA has the capacity to prevent metabolic disturbances in the brain [71]. On the other hand, it may be related to inflammation representing microglia activation because this is where *Glut*-*5* is primarily expressed in the central nervous system [72] as a constituent of the microglia “sensome” of genes [73].

## 5. Conclusions

Female *APOE4* carriers are at greater risk of AD, which may in part be explained by a menopause–*APOE4* interaction. The results of this study suggest that FO supplementation at 2 g/day attenuates multiple deleterious processes in the brain, ameliorating the impact of *APOE4* and menopause. As such, FO supplementation may offer a viable strategy to mitigate the deleterious *APOE4*–menopause interaction. Further investigation is warranted to establish if this is the case in humans.

## Figures and Tables

**Table 1:** Brain fatty acid composition.

Fatty Acid % Total	*APOE3*		*APOE4*				
	HF VCD	HF VCD FO	HF VCD	HF VCD FO	Genotype	Intervention	Interaction
Total n-3 PUFA	11.6 ± 0.70	13.6 ± 0.40	11.8 ± 0.50	13.5 ± 0.50	0.9991	**0.0022**	0.8036
20:5 n-3 (EPA)	0.03 ± 0.01	0.06 ± 0.03	0.03 ± 0.01	0.09 ± 0.03	0.9242	0.3191	0.2855
22:5 n-3	0.10 ± 0.05	0.07 ± 0.03	0.06 ± 0.02	0.04 ± 0.01	0.2796	0.3508	0.9236
22:6 n-3 (DHA)	11.2 ± 0.50	13.5 ± 0.40	11.7 ± 0.50	13.4 ± 0.50	0.7449	**0.0006**	0.5554
Total n-6	12.6 ± 0.40	11.1 ± 0.30	13.2 ± 0.60	11.5 ± 0.40	0.2726	**0.0036**	0.8050
18:2 n-6	0.43 ± 0.07	0.43 ± 0.04	0.39 ± 0.06	0.43 ± 0.05	0.7696	0.7616	0.6878
20:2 n-6	0.08 ± 0.03	0.10 ± 0.05	0.07 ± 0.02	0.09 ± 0.02	0.7328	0.6002	0.9964
20:3 n-6	0.21 ± 0.02	0.42 ± 0.03	0.23 ± 0.02	0.40 ± 0.05	0.9581	**<0.0001**	0.5492
20:4 n-6 (AA)	8.10 ± 0.39 b	7.5 ± 0.28 b	8.67 ± 0.50	7.88 ± 0.26	0.2471	0.0892	0.7692
22:4 n-6	3.23 ± 0.14	2.57 ± 0.07	3.07 ± 0.15	2.64 ± 0.15	0.7537	**0.0005**	0.3814
22:5 n-6	0.54 ± 0.09	0.02 ± 0.01	0.77 ± 0.10	0.03 ± 0.02	0.1017	**<0.0001**	0.1236
DHA:AA	1.39 ± 0.05	1.79 ± 0.02	1.36 ± 0.03	1.76 ± 0.04	0.3425	**<0.0001**	0.9501
SFA	35.5 ± 0.90	37.0 ± 0.60	36.9 ± 1.20	36.6 ± 0.80	0.5870	0.5145	0.3146
14:0	0.24 ± 0.04	0.09 ± 0.03	0.18 ± 0.05	0.10 ± 0.03	0.5783	**0.0052**	0.2971
16:0	14.70 ± 0.70	16.40 ± 0.40	17.30 ± 0.90	16.30 ± 0.70	0.1000	0.6241	0.0809
18:0	18.90 ± 0.50	19.60 ± 0.30	18.30 ± 0.60	19.40 ± 0.30	0.4033	0.0600	0.6637
20:0	0.41 ± 0.03	0.38 ± 0.02	0.33 ± 0.03	0.35 ± 0.04	0.0966	0.7664	0.4637
22:0	0.38 ± 0.05	0.25 ± 0.09	0.19 ± 0.06	0.25 ± 0.09	0.2206	0.7141	0.2283
24:0	0.78 ± 0.18	0.26 ± 0.13	0.53 ± 0.14	0.39 ± 0.11	0.6621	**0.0285**	0.1980
MFA	30.5 ± 0.8	30.0 ± 0.8	27.9 ± 1.4	30.2 ± 0.9	0.2229	0.1262	0.0984
16:1 n-9	0.22 ± 0.06	0.36 ± 0.05	0.37 ± 0.06	0.37 ± 0.06	0.1805	0.2057	0.2327
16:1 n-7	0.42 ± 0.01	0.48 ± 0.04	0.46 ± 0.04	0.50 ± 0.02	0.4596	0.1600	0.6908
18:1 n-9	21.20 ± 0.80	20.60 ± 0.40	19.30 ± 1.20	20.20 ± 0.40	0.1337	0.8116	0.3281
18:1 n-7	3.58 ± 0.16	3.77 ± 0.12	3.39 ± 0.13	3.59 ± 0.11	0.1694	0.1470	0.9972
20:1 n-9	3.04 ± 0.19	2.73 ± 0.16	2.21 ± 0.21	2.82 ± 0.31	0.1248	0.5351	0.0603
22:1 n-9	0.21 ± 0.03	0.18 ± 0.04	0.18 ± 0.02	0.18 ± 0.04	0.7174	0.6213	0.6748
24:1 n-9	1.96 ± 0.18 a	1.90 ± 0.16 a	1.14 ± 0.24 b	2.03 ± 0.23 a	0.1094	0.058	**0.0345**
Total DMA	9.82 ± 0.47	8.23 ± 0.51	8.61 ± 0.44	8.48 ± 0.72	0.9861	0.1982	0.1098

PUFA, polyunsaturated fatty acid; 20:5 n-3 EPA, eicosapentaenoic acid; 22:5 n-3, docosapentaenoic acid; 22:6 n-3 DHA, docosahexaenoic acid; 18:2 n-6,, linoleic acid; 20:2 n-6, eicosadienoic acid; 20:3 n-6, dihomo-gamma-linolenic acid; 20:4 n-6 AA, arachidonic acid; 22:4 n-6, adrenic acid; 22:5 n-6, docosapentaenoic acid; DHA:AA, docosahexaenoic acid to arachidonic acid ratio; SFA, saturated fatty acid; 14:0, myristic acid; 16:0, palmitic acid; 18:0, stearic acid; 20:0, eicosanoic acid; 22:0, docosanoic acid; 24:0, tetracosanoic acid; MUFA, monounsaturated fatty acid; 16:1 n-9, palmitoleic acid; 16:1 n-7, palmitoleic acid; 18:1 n-9, oleic acid; 18:1 n-7, cis-vaccenic acid; 20:1 n-9, 11-eicosenoic acid; 22:1 n-9, erucic acid; 24:1 n-9, nervonic acid. Brain fatty acid composition of experimental animals (*n* = 5/6 per group) Data is % of total fatty acids and mean value ± SEM. Two-way ANOVA; a,b denote significant differences as analysed via Benjamini–Hochberg FDR correction if interaction effect was established. Bold numbers show significant *p* values.

**Table 2:** RT-qPCR analysis of key bioenergetic and blood–brain barrier related genes in the hippocampus.

Gene	Category	*APOE3*		*APOE4*		Genotype	Intervention	Interaction
		HF VCD	HF VCD FO	HF VCD	HF VCD FO			
ERα	Oestrogen receptor	1.04 ± 0.15	0.91 ± 0.12	1.04 ± 0.15	1.06 ± 0.09	0.5836	0.6634	0.5687
Zo-1	Tight Junction	1.04 ± 0.13	0.92 ± 0.05	1.30 ± 0.12	0.97 ± 0.12	0.1762	0.0669	0.3045
Glut-1	Metabolic/Bioenergetic	1.01 ± 0.06	0.80 ± 0.06	0.87 ± 0.06	0.93 ± 0.08	0.9528	0.2605	0.0610
Glut-3	Metabolic/Bioenergetic	1.01 ± 0.08	0.801 ± 0.09	1.11 ± 0.07	0.94 ± 0.08	0.1463	**0.0243**	0.8017
Chrebp	Metabolic/Bioenergetic	1.01 ± 0.08	0.81 ± 0.08	1.11 ± 0.04	0.81 ± 0.06	0.4712	**0.0018**	0.4648
Gsk3b	Metabolic/Bioenergetic	1.01 ± 0.06	0.80 ± 0.06	1.12 ± 0.04	0.81 ± 0.10	0.3549	**0.0012**	0.4690
Aldob	Metabolic/Bioenergetic	1.04 ± 0.13	0.81 ± 0.11	1.4 ± 0.11	1.01 ± 0.10	**0.0237**	**0.0132**	0.4912

Hippocampal RT-qPCR of key genes (*n* = 5/6 per group). Data is presented as mean ΔΔCt value ± SEM. Results were analysed by two-way ANOVA. Bold numbers show significant *p* values.

## Data Availability

Data available upon request.

## Supplementary Materials

The following are available online at https://www.mdpi.com/article/10.3390/nu14091698/s1, Table S1: Full dietary information, Table S2: Primer sequences used for qPCR analysis, Table S3: Full correlation analysis results, Figure S1: Pearson’s correlation indicates that NOR behavioural test was not influenced by locomotor activity.
