# Supplementary material for: DHA-Enriched Fish Oil Ameliorates Deficits in Cognition Associated with Menopause and the APOE4 Genotype in Rodents
Source: Nutrients. 2022 Apr 19;14(9):1698. doi: 10.3390/nu14091698 (PMC9103304; doi:10.3390/nu14091698)
Supplement: Supplementary file 1 [file nutrients-14-01698-s001.zip › nutrients-1660594-supplementary.pdf]

Supplementary figures and tables to:

DHA-enriched fish oil ameliorates deficits in cognition associated with menopause and the APOE4 genotype in rodents

Matthew G. Pontifex[1*], Anneloes Martinsen[1], N. M. Saleh[1], Glenn Harden[1], Chris Fox[1,2], Michael Muller[1], David Vauzour[1#], Anne-Marie Minihane[1#]

1Norwich Medical School, University of East Anglia, Norwich, UK 1; m.pontifex@uea.ac.uk, a.martinsen@uea.ac.uk, r.saleh@uea.ac.uk, g.harden@uea.ac.uk , chris.fox@uea.ac.uk, michael.muller@uea.ac.uk d.vauzour@uea.ac.uk, , a.minihane@uea.ac.uk

2Exeter Medical School, University of Exeter, Exeter, UK; Christopher.fox@exeter.ac.uk

**To whom correspondence should be addressed*

Matthew Pontifex, University of East Anglia, Norwich Medical School, Norwich NR4 7UQ, United Kingdom.

Email: M.Pontifex@uea.ac.uk

Table S1: Full dietary composition of diet used in the experimentations.

Diets	High Fat (HF)	High fat - High fish oil (HF FO)
**% Composition**	**Kcal**	**Kcal**
Protein	20	20
Carbohydrate	35	35
Fat	45	45
Total	100	100
**Diet component gm/kg**	**gm/kg**	**gm/kg**
Casein	233.0	233.1
L-Cystine	3.5	3.5
Corn starch	84.8	84.8
Maltodextrin 10	116.5	116.5
Sucrose	201.4	201.4
Cellulose, BW200	58.3	58.3
Corn oil	47.2	46.3
Palm oil	188.8	185.0
EPAX oil	0	4.7
Mineral mix S10026	11.7	11.7
Dicalcium Phospate	15.2	15.1
Calcium Carbonate	6.4	6.4
Potassium Citrate, 1 H20	19.2	19.2
Vitamin mix V10001	11.7	11.7
Choline Bitartrate	2.3	2.3

Table S2 Primer sequences used for qRT-PCR

Gene	Description	Forward Primer (5' to 3')	Reverse Primer (5' to 3')
Aldob	Aldolase B	CTGTGTTGAGGATTGCTGACCAG	TCAGGAAGCACCTCTGGCTCAA
Chrebp	Carbohydrate response element binding protein	GAGTGCTTGAGCCTGGCTTACA	GCTCTCCAGATGGCGTTGTTCA
Cldn1	Claudin 1	GGGGACAACATCGTGACCG	AGGAGTCGAAGACTTTGCA
ERα	Estrogen receptor alpha	AATTCTGACAATCGACGCCAG	GTGCTTCAACATTCTCCCTCCTC
ERβ	Estrogen receptor beta	TTCCCGGCAGCACCAGTAACC	TCCCTCTTTGCGTTTGGACTA
Gapdh	Glyceraldehyde 3-phosphate dehydrogenase	AGGTCGGTGTGAACGGATTTG	TGTAGACCATGTAGTTGAGGTCA
Glut-1	Glucose transporter 1	GCTTCTCCAACTGGACCTCAAAC	ACGAGGAGCACCGTGAAGATGA
Glut-3	Glucose transporter 3	CCGCTTCTCATCTCCATTGT	CCTGCTCCAATCGTGGCATAGA
Glut-5	glucose transporter 5	CCAATATGGGTACAACGTAGCTG	GCGTCAAGGTGAAGGACTCAATA
Gsk3b	Glycogen Synthase Kinase 3 Beta	GAGCCACTGATTACACGTCCAG	CCAACTGATCCACACCACTGTC
Nfkb1	Nuclear Factor Kappa B Subunit 1	GGCAGGCTATTGCTCATCACAG	GCTGCCAAAGAAGGACACGACA
ZO-1	Zonula occludens-1	CCACCTCTGTCCAGCTCTTC	CACCGGAGTGATGGTTTTCT

Table S3: Typical Fatty Acid Profile of HF diet

Fatty acid	(gm/4057 kcal)
C10, Capric	0.107
C12, Lauric	0.163
C14, Myristic	2.1
C15	0.14
C16, Palmitic	36.8
C16:1, Palmitoleic	2.5
C17	0.675
C18, Stearic	19.8
C18:1 Oleic	64.1
C18:2 Linoleic	56.2
C18:3Linolenic	4.2
C20 Arachidic	0.4
C20:1	1.16
C20:2	1.4
C20:3	0.2
C20:4 Arachidonic	0.5
C22, Behenic	0.1
C22:5 Docosapentaenoic	0.152

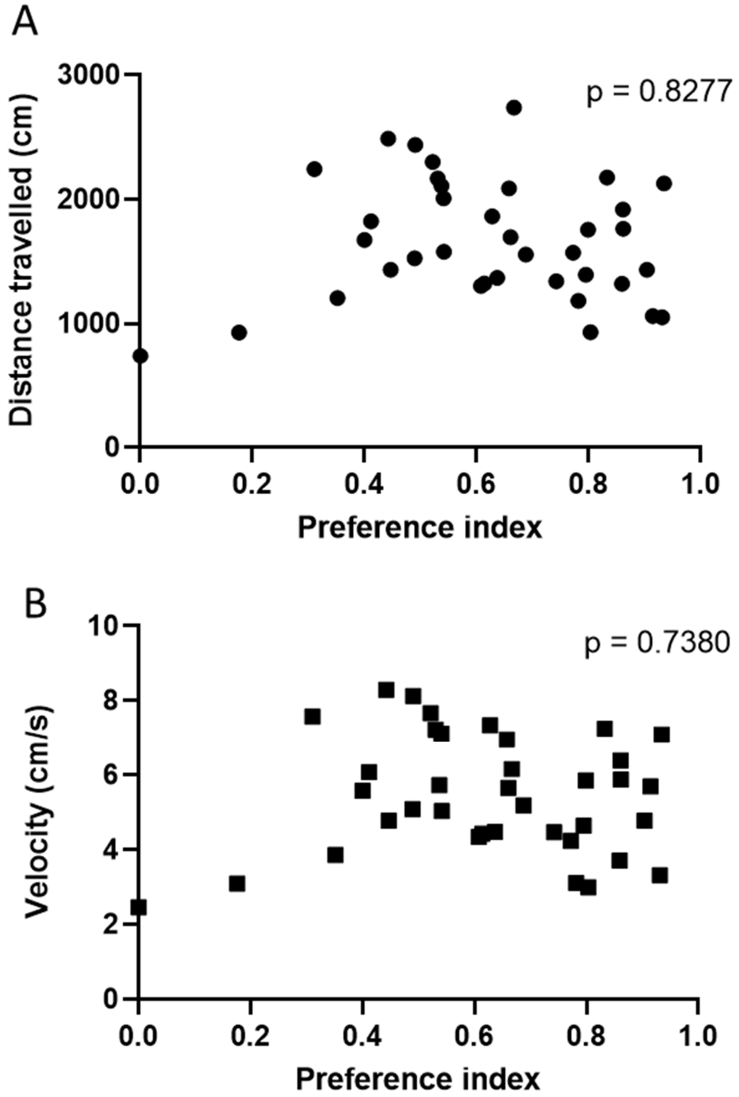

Figure S1: Pearson's correlation indicates that NOR behavioural test was not influenced by locomotor activity. (**A**). Distance travelled; (**B**) Velocity.
